# Readiness assessment: Implications for COVID-19 infection prevention and control (IPC) preparedness in health facilities

**DOI:** 10.1017/ash.2022.207

**Published:** 2022-05-16

**Authors:** Evelyn Wesangula, Veronica Kamau, Felister Kiberenge, Emmanuel Tanui, Susan Githii, Linus Ndegwa, George Owiso, Irungu Kamau, Jennifer Njuhigu

## Abstract

**Background:** Monitoring uptake of infection prevention and control (IPC) interventions is critical for the targeted and rational use of limited resources. A national facility readiness assessment conducted in August 2020 provided key information for targeted interventions to strengthen priority IPC areas. We assessed the level of COVID-19 preparedness in the facilities, identified priority COVID-19 IPC gaps, and generated a baseline report to further guide IPC investments at all levels. **Methods:** The Kenya Ministry of Health in collaboration with the CDC and International Training and Education Center for Health adapted a WHO Facility Readiness Assessment tool to include COVID-19–specific areas. In August 2020, data were collected using tablets through an Android-based electronic platform and were analyzed using descriptive statistics. Assessments were conducted in public, private, and faith-based health facilities nationally after 4 months of preparedness and investment in the healthcare system. **Results:** We assessed 684 facilities of the targeted 844 (81%). Overall facility readiness in Kenya was rated above average (61%), and the performance score significantly increased with the Kenya Essential Package for Health level, with level 5 and 6 facilities scoring an average of 83% and 79% respectively. Of the assessed facilities, 82% had an appointed IPC coordinator. Only 14% of the facilities had all the required guidelines, policies, and the appropriate COVID-19 case definitions. 67% of the facilities had updated supply inventories for past week. Only 50% of the facilities had adequate supplies of N95 masks. The assessment revealed that 52% of healthcare facilities had trained their healthcare workforce; morticians were the least trained (only 17% of facilities). Moreover, 41% of the facilities had clear work plans for monitoring healthcare workers exposures to COVID-19, but only 33% of the facilities had policies on the management of infected healthcare workers. **Conclusions:** The findings provided critical information for stakeholders at all levels to be used for policy decisions, to prioritize key intervention areas in leadership and governance of facility IPC programs, for guideline development, and for capacity building and targeted investment in IPC to improve COVID-19 facility preparedness.

**Funding:** None

**Disclosures:** None

